# Examining intra-rater and inter-rater response agreement: A medical chart abstraction study of a community-based asthma care program

**DOI:** 10.1186/1471-2288-8-29

**Published:** 2008-05-09

**Authors:** Teresa To, Eileen Estrabillo, Chengning Wang, Lisa Cicutto

**Affiliations:** 1 Child Health Evaluative Sciences, Research Institute, The Hospital for Sick Children, Toronto, Ontario, Canada; 2 Pediatrics, The Hospital for Sick Children, Toronto, Ontario, Canada; 3 Respiratory Medicine, The Hospital for Sick Children, Toronto, Ontario, Canada; 4 The University of Toronto, Toronto, Ontario, Canada

## Abstract

**Background:**

To assess the intra- and inter-rater agreement of chart abstractors from multiple sites involved in the evaluation of an Asthma Care Program (ACP).

**Methods:**

For intra-rater agreement, 110 charts randomly selected from 1,433 patients enrolled in the ACP across eight Ontario communities were re-abstracted by 10 abstractors. For inter-rater agreement, data abstractors reviewed a set of eight fictitious charts. Data abstraction involved information pertaining to six categories: physical assessment, asthma control, spirometry, asthma education, referral visits, and medication side effects. Percentage agreement and the kappa statistic (κ) were used to measure agreement. Sensitivity and specificity estimates were calculated comparing results from all raters against the gold standard.

**Results:**

Intra-rater re-abstraction yielded an overall kappa of 0.81. Kappa values for the chart abstraction categories were: physical assessment (κ 0.84), asthma control (κ 0.83), spirometry (κ 0.84), asthma education (κ 0.72), referral visits (κ 0.59) and medication side effects (κ 0.51). Inter-rater abstraction of the fictitious charts produced an overall kappa of 0.75, sensitivity of 0.91 and specificity of 0.89. Abstractors demonstrated agreement for physical assessment (κ 0.88, sensitivity and specificity 0.95), asthma control (κ 0.68, sensitivity 0.89, specificity 0.85), referral visits (κ 0.77, sensitivity 0.88, specificity 0.95), and asthma education (κ 0.49, sensitivity 0.87, specificity 0.77).

**Conclusion:**

Though collected by multiple abstractors, the results show high sensitivity and specificity and substantial to excellent inter- and intra-rater agreement, assuring confidence in the use of chart abstraction for evaluating the ACP.

## Background

Medical chart abstraction is a common method of data collection in clinical and health care epidemiologic studies [1]. In a review of emergency medicine research articles, 244 of 986 primary studies (25%) relied on chart reviews [2]. Beard et al also used more than 18,000 medical records to conduct a retrospective descriptive study of asthma prevalence [3]. The extensive use of chart abstraction highlights the importance of assessing the intra-rater and inter-rater reliability of the collected data, as it reflects the quality of the data and the value of the results [4].

Although many investigators have expressed concern regarding the reliability associated with data abstracted from medical records [1,2,5,6], few studies report details regarding the types of data elements that were assessed and the methods used to ensure reliability [2,7-9]. For multi-centre studies, the need for reliability assessments is especially important given the involvement of multiple data abstractors and the potential for variability. Thus, designing a reliability assessment study that uses appropriate sampling techniques and analytic methods offers an efficient way to verify the reliability of the data collected through chart review. Indications of high agreement can provide confidence in the data collection process and the subsequent conclusions drawn from those data [10].

Previous studies have assessed the reliability of medical record reviews in the context of screening and the detection of adverse events [11,12]. This paper describes a secondary chart abstraction study carried out as part of the multi-site Primary Care Asthma Pilot Project (PCAPP) to determine the effectiveness of an evidence-based community asthma care program. The purpose of chart re-abstraction was to measure the intra- and inter-rater reliability of abstracted patient chart data across sites and assessors involved in PCAPP.

## Methods

### Primary Care Asthma Pilot Project (PCAPP)

The Primary Care Asthma Pilot Project (PCAPP) was a community-based participatory study funded by the Ontario Ministry of Health and Long-Term Care. Initiated in 2003, PCAPP was designed to determine whether the use of an evidence-based Asthma Care Program (ACP) would lead to improved asthma care delivery and outcomes for patients from 15 satellite clinics in eight local communities across Ontario. Patients included in PCAPP were those aged 2 to 55 with mild to moderate asthma. The satellite clinics included eight Community Health Centres, a Rural Family Health Team, a Group Health Centre and an Aboriginal Access Centre. Participants in the PCAPP project consented to have their medical charts reviewed on four occasions to measure the process of care involved in the implementation of the ACP. Ten different research staff conducted chart abstraction across the various sites, thus it was essential to ensure that data were collected consistently over time within each participating site (intra-rater reliability) and across sites (inter-rater reliability).

### Initial data collection and reabstraction

The original chart extraction and coding of the 1,433 PCAPP participants' records occurred between September 2003 and June 2005. Information was abstracted on prospective patient visits that took place at baseline, 6-month follow-up, and 12-month follow-up to measure the process of care during the implementation of the ACP. Information was also abstracted from retrospective patient visits that happened between January 1, 2002 and December 31, 2002 in order to describe the pattern of asthma care prior to the ACP implementation. Data from patient medical records were collected using a data abstraction form containing 33 items grouped into six categories (Table 1). Abstractors indicated on the form whether information pertaining to each item was "documented" or "not documented" in the patient's chart. The chart abstraction study and the accompanying form were approved by the Research Ethics Board at the Hospital for Sick Children, Toronto.

**Table 1:** Categories and items for chart abstraction

**Categories and items**	
***1. Physical assessment***	***4. Asthma education***
1.1. Temperature	4.1. Review of asthma definition
1.2. Pulse	4.2. Provision of an asthma action plan
1.3. Respiratory rate	4.3. Verbal review of action plan
1.4. Blood pressure	4.4. Review of proper medication techniques
1.5. Height	4.5. Review of warning signs
1.6. Weight	4.6. Review of asthma triggers
1.7. Chest sounds	4.7. Review of control measures
1.8. Oxygen saturation	4.8. Management and coping strategies
***2. Asthma control***	***5. Asthma referrals***
2.1. Cough	5.1. Asthma education programs
2.2. Waking at night	5.2. Asthma support groups
2.3. Physical activity limitations	5.3. Specialists
2.4. Reliever use with exercise	
2.5. Reliever use < 4 times per week	
2.6. Exacerbations (hospital, ED visits)	
2.7. School or work absenteeism	
***3. Spirometry***	***6. Medications***
3.1. FEV1 pre-test	6.1. Side effects
3.2. FEV1 post-test	
3.3. FEV1 % change	
3.4. PEF pre-test	
3.5. PEF post-test	
3.6. PEF % change	Total number of items = 33

Abbreviations: ED emergency department, FEV1 Forced Expiratory Volume in 1 second, PEF peak expiratory flow

In the current study, assessment of chart abstraction reliability involved distinct methods of comparison for intra-rater agreement and for inter-rater agreement. *Intra-rater agreement *was based on the re-abstraction of medical charts by abstractors from the same site at Time 2 (between July 2005 and February 2006) and comparing the re-abstracted data to data collected at the initial abstraction at Time 1 (between September 2003 and June 2005). To minimize the potential for recall and artificial inflation of observed agreement, all data abstracted during Time 1 (initial chart abstraction) were returned to the research centre upon their completion, and chart abstractors were not granted access to their original responses at Time 2 (chart re-abstraction). *Inter-rater agreement *involved comparisons between abstractors and a gold standard using a single set of eight fictitious medical charts at the end of the study.

### Definition of chart abstraction items

Six chart abstraction categories were used as a basis for determining chart abstractor agreement: physical assessment, asthma control, spirometry, asthma education, referrals to specialists or education programs, and medications prescribed. The chart abstraction form included space for abstractors to record specific medication information. However, details such as generic/trade name, strength, dosing, route of administration and duration were considered especially challenging to analyze quantitatively. Thus for the purpose of this study, only the presence of medication side effects was included as a dichotomous yes/no variable for agreement analyses. A summary of the number of chart abstraction items within each category is shown in Table 1.

### Data quality control measures

As part of the main study, site coordinators and chart abstractors took part in training workshops and orientation sessions to introduce the project and research methodology to the local staff. Chart abstraction guides were distributed to all sites and included abstraction procedures, coding instructions, and several scenarios for discussion so that chart abstractors would handle potentially challenging medical charts in a consistent manner. Additional site visits were conducted three to five months following the implementation of PCAPP to review data collection processes and to ensure that research protocols were being followed. Other ongoing communication methods included email, teleconferencing, and regularly scheduled Advisory Committee meetings.

Following abstraction, chart abstractors entered the data into a database using Microsoft Access (Microsoft Corporation, Redmond, Washington). Data were transferred monthly between the participating sites and the lead investigating centre, where data were tracked and cleaned.

### Assessing intra-rater reliability

To assess *intra-rater reliability*, ten abstractors re-abstracted data at Time 2 from randomly selected patient charts that had been abstracted at Time 1. A sample of 110 patient charts representing 8% of the main study population was randomly selected from the original sample for re-abstraction. Random number generation and subsequent analyses were conducted using SAS Statistical Software version 9 [13]. The formula given by Sim and Wright [14], indicated that the sample size for this re-abstraction study would allow the detection of a kappa statistic between the values 0.60 and 0.70 with 80% power at an alpha level of 0.05.

### Assessing inter-rater reliability

**Figure 1:**
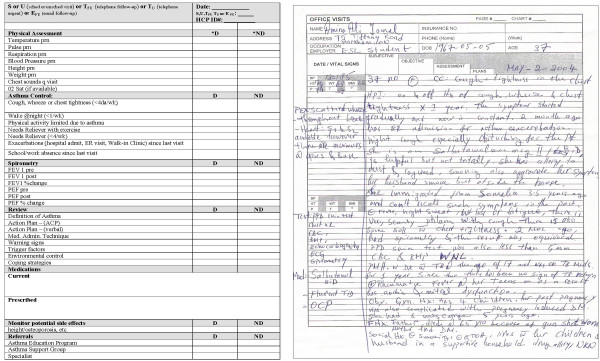
Chart abstraction form and sample portion of a fictitious medical chart used for assessing inter-rater reliability.

To assess inter-rater agreement while ensuring that patient charts remained in their respective sites to safeguard patient confidentiality, an innovative method was used wherein 8 fictitious medical charts were distributed to each site chart abstractor as well as an experienced chart abstractor who is not involved in the study. All abstractors abstracted information from the eight simulated charts using the study chart abstraction form. The simulated charts reflected a range of patient characteristics: young versus old, few versus multiple co-morbidities, controlled versus uncontrolled asthma. Pilot testing of the fictitious medical charts for face and content validity occurred at two sites by individuals not involved in chart abstraction. The set of charts underwent minimal revision. Examples of the simulated patient chart and the chart abstraction form are illustrated in Figure 1.

### Analysis

Intra-rater reliability was assessed using the overall percentage of agreement [15], and the kappa statistic. The abstractors' responses given during the initial abstraction (Time 1) were compared to those provided during the re-abstraction (Time 2). To ensure proper comparison, the abstracted data were matched by study identification numbers and corresponding asthma-related visit dates.

Percentage agreement and the kappa statistic were also used to assess inter-rater reliability across the study sites for the eight simulated patient charts. Data abstractions from the simulated charts by site abstractors were checked against the data abstracted by the experienced chart abstractor, who served as a gold standard. Sensitivity and specificity estimates were calculated comparing results from all raters against the gold standard.

Using the algorithm of Landis and Koch [16], kappa values of 0.80 and above represented excellent agreement, values between 0.61 and 0.80 represented substantial agreement, 0.41 to 0.61 represented moderate agreement, and values below 0.40 suggested fair to poor agreement.

Summary kappa scores and percentage agreement were calculated for each category (n = 6) and also for each variable (n = 33). Agreement coefficients for each of the variables were calculated to determine the range of agreement within each category. The overall kappa score was calculated by summation of scores within each category, and subsequently using the category-specific kappa values to compute an overall kappa summary statistic. The homogeneity of kappas across chart abstraction categories was tested using the method outlined by Donner et al [17].

## Results

### Intra-rater agreement

For the intra-rater component of the re-abstraction study, 110 charts were reviewed, and 218 documented asthma-related patient visits were included in comparing chart abstraction at Time 1 and Time 2.

The overall intra-rater percentage of agreement and kappa statistics were 93% and 0.81 respectively. Across the six categories, kappa varied between 0.51 and 0.84, with categories pertaining to asthma referrals and medication side effects showing only moderate agreement. Table 2 presents a summary of the results for category-specific and overall measures of agreement for intra-rater reliability. Homogeneity testing of the category-specific kappa values suggested statistically significant heterogeneity. It was therefore of interest to explore possible sources of heterogeneity. Data were pooled and subsequently stratified by abstractor to compare the degree of agreement between Time 1 and Time 2 for each individual abstractor (Table 3). The abstractor-specific percentage agreement varied from 79% to 98%, while the abstractor-specific intra-rater kappa statistics ranged from 0.21 to 0.94. The heterogeneity chi-square statistic for the difference in the kappas calculated for the 10 abstractors was also statistically significant (χ^2 ^= 238, p < 0.0001), suggesting that individual differences in abstractor consistency may account for the heterogeneity of the overall category-specific kappa values. Intra-rater agreement was also examined for all the abstractors according to when the chart was abstracted, either at the start of the study (on retrospective patient visits prior to baseline) or at 6-month follow-up. The kappa statistics were 0.76 (95% CI: 0.73, 0.79) for retrospectively abstracted charts, and 0.82 (95% CI: 0.80, 0.85) for 6-month follow-up chart abstraction (data not shown in tables).

**Table 2:** Category-specific and overall intra-rater reliability coefficients

	Percentage agreement		Kappa		
		
Category (number of items)	Category %	% range across items	Category κ	95% CI	κ range across items
1. Physical assessment (8)	94	91–98	0.84	(0.81–0.87)	0.72–0.90
2. Asthma control (7)	92	85–95	0.83	(0.80–0.86)	0.60–0.88
3. Spirometry (6)	95	91–97	0.84	(0.80–0.86)	0.78–0.94
4. Asthma education (8)	91	78–95	0.72	(0.68–0.76)	0.55–0.82
5. Asthma referrals (3)	94	92–93	0.59	(0.46–0.71)	0.44–0.65
6. Medications (1)	94	94	0.51	(0.27–0.75)	0.51
Overall	93		0.81*	(0.80–0.83)	

* Chi-square test statistic for homogeneity = 47, df = 5, p < 0.0001

Abbreviations: κ kappa statistic, CI confidence interval

**Table 3:** Abstractor-specific intra-rater reliability coefficients

		Kappa*	
		
Abstractors	Percentage agreement (%)	κ	95% CI
1	88	0.21	(0.02–0.40)
2	85	0.71	(0.62–0.80)
3	91	0.79	(0.74–0.84)
4	94	0.83	(0.79–0.87)
5	96	0.89	(0.85–0.92)
6	94	0.78	(0.66–0.89)
7	93	0.78	(0.72–0.84)
8	79	0.57	(0.50–0.63)
9	88	0.66	(0.57–0.75)
10	98	0.94	(0.92–0.96)

* Chi-square test statistic for homogeneity = 238, df = 9, p < 0.0001

Abbreviations: κ kappa statistic, CI confidence interval

### Inter-rater agreement

As expected, inter-rater agreement measures were slightly lower than those for intra-rater agreement. The overall inter-rater percentage of agreement and kappa statistics were 88% and 0.76 respectively (Table 4). Kappa values showed moderate agreement for the category of asthma education, and could not be calculated for the spirometry and medication side effects categories due to a high observed percentage of agreement. The overall sensitivity and specificity estimates across all six categories were 0.91 and 0.89 respectively (Table 4), which indicate consistent abstractions conducted by the raters in comparison to the gold standard.

**Table 4:** Category-specific and overall inter-rater reliability coefficients, sensitivity and specificity

	Percentage agreement		Kappa		Sensitivity^§^	Specificity^§^
				
Category (number of items)	Category %	% range across items	Category κ (95% CI)	κ range across items	Estimate (95% CI)	Estimate (95% CI)
1. Physical assessment (8)	95	79–100	0.88 (0.87–0.90)	0.00–1.00	0.95 (0.92–0.97)	0.95 (0.91–0.98)
2. Asthma control (7)	84	76–93	0.68 (0.65–0.70)	0.05–0.71	0.89 (0.84–0.92)	0.85 (0.80–0.88)
3. Spirometry (6)	100	100	nc	nc	nc	0.99 (0.98–1.00)
4. Asthma education (8)	76	54–95	0.49 (0.46–0.52)	0.04–0.67	0.87 (0.80–0.92)	0.77 (0.73–0.81)
5. Asthma referrals (3)	90	88–93	0.77 (0.73–0.81)	0.02–0.84	0.88 (0.77–0.95)	0.95 (0.90–0.98)
6. Medications (1)	90	90	nc	nc	nc	0.94 (0.85–0.98)
Overall	88		0.75* (0.75–0.77)		0.91 (0.89–0.93)	0.89 (0.88–0.91)

* Chi-square test statistic for homogeneity = 737, df = 4, p < 0.0001

§ Sensitivity and specificity are calculated to compare assessments of all raters against the gold standard.

Abbreviations: κ kappa statistic, CI confidence interval, nc not calculable

**Figure 2:**
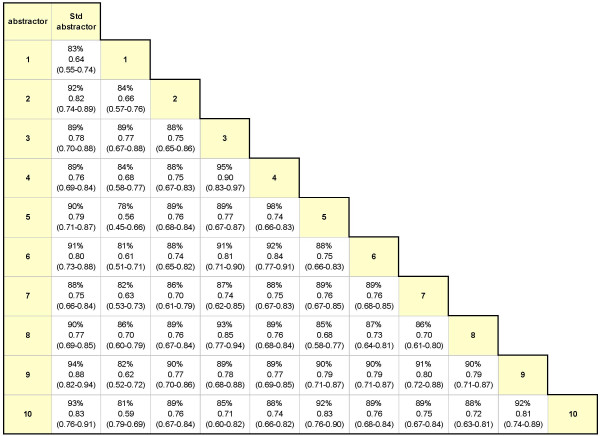
**Ranges of inter-rater agreement among pairs of abstractors**. Data are shown as percentage agreement (%), kappa coefficient, and 95% confidence interval for kappa. Abbreviations: Std = Gold Standard.

The inter-rater kappa values were shown to be statistically heterogeneous according to the homogeneity test (χ^2 ^= 737, p < 0.0001). To explore the source of heterogeneity, data abstracted from the eight simulated charts were pooled and agreement coefficients were calculated for each paired combination of abstractors including the standard abstractor who was not involved in the study. The range of percentage agreement and kappa values are shown in Figure 2. The percentage agreement across the different pairs of abstractors ranged from 78% to 98%. Kappa values ranged from 0.56 to 0.90 and were statistically heterogeneous (χ^2 ^= 128 on 54 degrees of freedom, p < 0.0001). Inter-rater agreement was then examined for each simulated chart. As shown in Table 5, the percentage agreement was consistent across the eight charts (85% to 91%), and kappa statistics ranged from 0.69 to 0.78. The sensitivity estimates ranged from 0.84 to 0.99 and specificity estimates ranged from 0.85 to 0.96.

**Table 5:** Simulated chart-specific inter-rater reliability coefficients

		Kappa*		Sensitivity^§^		Specificity^§^	
				
Simulated Charts	Percentage agreement (%)	κ	95% CI	Estimate	95% CI	Estimate	95% CI
1	88	0.75	(0.72–0.78)	0.92	(0.86–0.96)	0.90	(0.85–0.94)
2	91	0.78	(0.75–0.82)	0.99	(0.91–1.00)	0.90	(0.85–0.93)
3	90	0.70	(0.66–0.74)	0.84	(0.73–0.91)	0.96	(0.92–0.98)
4	88	0.76	(0.73–0.79)	0.96	(0.90–0.99)	0.87	(0.82–0.91)
5	88	0.74	(0.71–0.78)	0.91	(0.82–0.96)	0.88	(0.83–0.92)
6	85	0.69	(0.65–0.73)	0.93	(0.85–0.97)	0.85	(0.79–0.90)
7	88	0.76	(0.73–0.79)	0.91	(0.85–0.95)	0.91	(0.85–0.95)
8	86	0.72	(0.69–0.76)	0.85	(0.77–0.90)	0.85	(0.79–0.90)

* Chi-square test statistic for homogeneity = 22, df = 7, p = 0.003

§ Sensitivity and specificity are calculated to compare assessments of all raters against the gold standard.

Abbreviations: κ kappa statistic, CI confidence interval

## Discussion

The results of this study showed that chart abstractors involved in data collection for the Primary Care Asthma Pilot Project reliably extracted information contained in the medical charts. Previous studies of intra-rater and inter-rater reliability have also demonstrated moderate to substantial intra-rater and inter-rater reliability associated with medical chart abstraction [6,18]. When abstracted by the same rater, or raters within the same centre, the majority of items (27 of 33, 82%) had kappa values greater than 0.61, suggesting that intra-rater agreement was substantial to excellent overall as well as on a per-item basis despite changes in staff that occurred at some sites. For inter-rater agreement, 10 of 33 items (30%) were associated with a kappa value greater than 0.61. Fewer items showed substantial inter-rater agreement as compared to intra-rater agreement, which may be attributable to the content of the fictitious charts used for inter-rater assessment rather than differences in the abstraction process or between the abstractors themselves.

Closer examination of the agreement coefficients revealed differences in kappa statistics depending on the chart abstraction category. For example, the kappa statistics indicated only moderate agreement for intra-rater data abstraction relating to asthma referrals and medication side effects. Moderate agreement was also shown for inter-rater data abstraction relating to asthma education. Information pertaining to these abstraction categories was presented in the medical charts as free-text or in narrative form. The demonstration of moderate agreement may be due to differences in the abstractors' familiarity with the concepts and terminology of asthma treatment strategies, the indications for specialist referrals, and how and where these details would be noted in the medical chart. Other studies have identified similar issues as having a potential impact on reliability [7,9,19]. Our findings showed that intra-rater reliability was significantly improved for 6-month follow-up chart abstraction compared to retrospective chart abstraction at baseline, which may be attributable to the effect of ongoing abstractor training and increasing familiarization with the nuances of chart abstraction over time.

A salient challenge of chart review is the accurate abstraction of medication information, dosing, frequency and duration. Quantifying agreement for the documentation of pharmaceutical treatments has important clinical implications. This study was unable to analyse treatment in this context. However, results showed moderate intra-rater and inter-rater agreement for the categories of asthma education, asthma referral, and medication side effects, suggesting that agreement for more specific medication regimens may be moderate at best. Further studies are needed to assess the agreement in medical chart documentation of treatments.

Results from this study also reflect the paradox associated with the kappa statistic wherein an item or category demonstrates high percentage agreement but a low kappa coefficient [20]. This inherent limitation of kappa is well-established and acknowledged. Thus, in some instances, the percentage agreement may be a more appropriate measure of reliability. In the presence of a reference or a gold standard, test statistics such as sensitivity, specificity, predictive values, and likelihood ratios are more often used than the simple kappa statistic. In this study, since a gold standard was available, it was possible to compute sensitivity and specificity estimates across all the categories and for all the charts created. The overall sensitivity and specificity measures in this study were in the order of 90% indicating good validity. Despite the limitations of the kappa statistic, it was also used to present results of the current study since it provides a simple measure to assess intra-rater reliability (precision) and also inter-rater reliability in studies that involve multiple raters. While there are other methods of assessing interobserver agreement, kappa remains by far the most commonly reported measure in the medical literature.

To address the objectives of this multi-site study, assessing chart abstraction agreement required the computation of multiple kappa statistics with the underlying assumption that these individual measures could be combined statistically into overall values for intra-rater reliability and inter-rater reliability. The overall results indicated substantial to excellent agreement though statistical tests suggested significant heterogeneity in the kappa statistics. Further investigation of possible sources of heterogeneity, including the clinical knowledge base of chart abstractors, may be relevant to enhancing the design of studies involving clinical interventions that are documented and subsequently abstracted from patient medical charts.

Although the overall intra- and inter-rater agreement was high in the current study, the reliability of chart abstraction data remained less than 100%. This means that there may still be some misclassification during data collection, which may result in a bias towards the null hypothesis (Type II error) of the intervention study. One way to overcome this potential problem is to obtain a large enough sample size. Future evaluations of chart abstraction may include regression modelling to investigate the heterogeneity in kappa statistics and the impact of possible explanatory factors on the probability of achieving agreement. Methods such as the generalized estimating equation (GEE) approach of Liang and Zeger [21] are considered useful for intra-rater agreement involving clustered, multi-centre designs where data are collected at multiple time periods. Based on the elements involved in the current study, possible covariates for investigation may include the number of abstractors involved, the differences in chart abstraction items or categories, and the duration of time between abstractions.

## Conclusion

This study demonstrates that chart abstraction can be a reliable form of data collection in multi-centre studies for asthma. The ongoing assessment of data reliability offers an effective way of monitoring data quality, which can subsequently improve the reliability of the results drawn from the data.

## Competing interests

The authors declare that they have no competing interests.

## Authors' contributions

TT designed and conducted the study, and was responsible for collecting the data, supervising the statistical analyses, interpreting the findings and writing this manuscript. EE conducted the statistical analyses and drafted the manuscript. LC provided critical revisions of the manuscript for important intellectual content. CW served as the gold standard in the chart abstraction study and provided critical revisions of the manuscript.

## Pre-publication history

The pre-publication history for this paper can be accessed here:


